# Extended spectral response of cavity-based terahertz photoconductive antennas and coherent detection of quantum cascade lasers

**DOI:** 10.1515/nanoph-2025-0088

**Published:** 2025-06-26

**Authors:** Anna De Vetter, James Normansell, José Palomo, Martin Mičica, Li Chen, Juliette Mangeney, Jerome Tignon, Lianhe H. Li, Alexander Giles Davies, Edmund H. Linfield, Joshua R. Freeman, Sukhdeep S. Dhillon

**Affiliations:** Laboratoire de Physique de l’Ecole Normale Supérieure (LPENS), 24 rue Lhomond 75005 Paris, France; School of Electronic and Electrical Engineering, University of Leeds, Leeds LS2 9JT, UK

**Keywords:** ultrafast terahertz detection, photoconductive antennas, cavities, time domain spectroscopy

## Abstract

Coherent ultrafast detection has become an important method to study the temporal response of terahertz (THz) quantum cascade lasers (QCLs), bringing insights into the dynamics of modelocking and frequency comb operation in these complex structures. Coherent detection has been typically based on the use of nonlinear crystals and electro-optic sampling, which are less sensitive to QCLs operating at high THz frequencies. This is because their response drops rapidly with frequency owing to phase matching conditions. Here, we develop coherent detection based on THz photoconductive antennas in vertical quarter-wavelength cavities, where we can freely engineer the spectral response to enhance the THz detection at frequencies greater than 2 THz. We develop thick low temperature grown GaAs that is transferred onto polymer and metal coated substrates to create a cavity with an THz response that exceeds the response of non-cavity detectors. This vertical THz cavity also permits the planar electrode geometry to be designed independently. Indeed, we show that the THz cavity can be combined with large surface area single contact electrodes to further enhance the spectral response. Although this proof-of-principle coherent detection is not fully optimised, it is used to coherently resolve the temporal response of a double-metal THz QCL operating at 3 THz. This approach opens up perspectives to tune the response of THz photoconductive antennas and enhance their spectral response at a desired frequency.

## 1 Introduction

The terahertz (THz) region of the electromagnetic spectrum presents remarkable opportunities for applications including spectroscopy, non-destructive imaging, ultra-fast communications, and the study of fundamental phenomena occurring in the THz range [1]. THz time domain spectroscopy (TDS) has become one of the most important methods that is applied in this spectral range [2], as it permits sensitive time resolved detection of ultrashort THz pulses. For example, precise measurements of the complex conductivity, absorption coefficient and refractive index of materials can be easily achieved [3]. THz TDS relies on ultrafast femtosecond lasers in the near-infrared that are used to coherently generate and detect phase-resolved THz pulses via nonlinear crystals or photoconductive antennas. Coherent THz detection techniques have also become important for THz quantum cascade lasers (QCLs), as they provide insights into the ultrafast temporal response of these sources. By retrieving the amplitude and phase of THz QCL emission, one can precisely reconstruct the electric field over time with femtosecond resolution and access key information on the behaviour of these complex lasers [4], [5]. This has become of great importance to demonstrate their ultrafast temporal response, permitting insights into, for example, frequency comb regime [6] in both frequency and amplitude modulated regimes [7], [8], [9] as well as ultrashort nonlinearities and pulse generation [10], [11], [12], where typical power detectors cannot access this information. However, current methods of coherent detection of QCL emission display a limited and un-optimised sensitivity.

**Figure 1:**
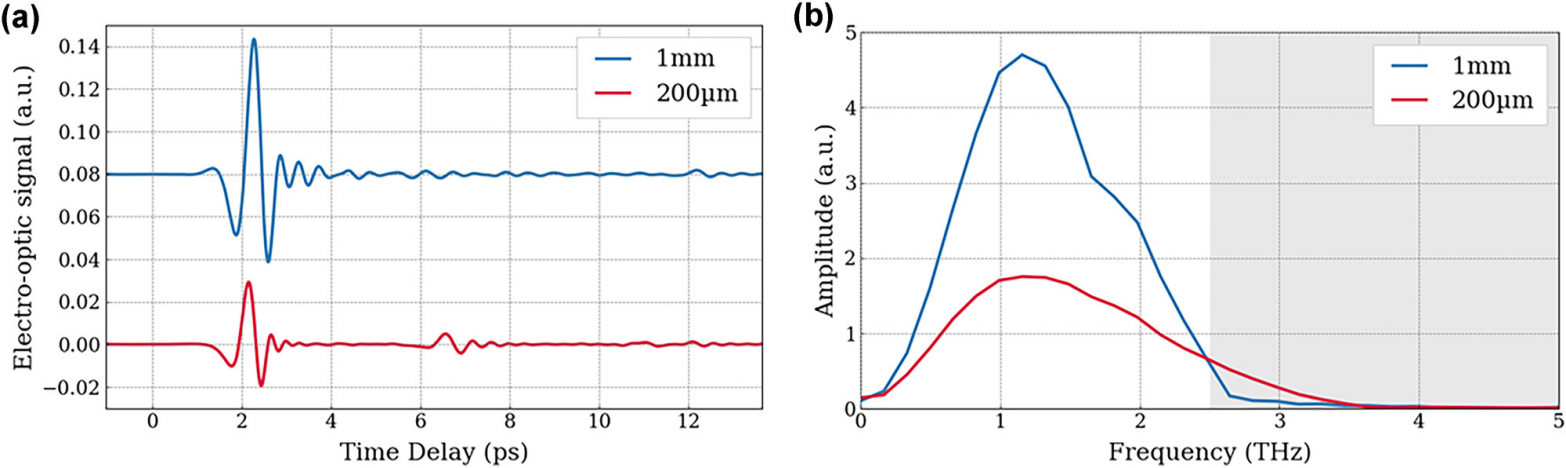
Influence of electro-optic sampling crystal thickness on THz detection. (a) Coherent THz pulse emitted by an interdigitated PCA and detected by electo-optic sampling with a 1 mm-thick ZnTe (blue line) and a 200 µm-thick ZnTe (red line) crystal. (b) Frequency spectrum corresponding to the FT of the time resolved electric field with 1 mm-thick ZnTe (blue line) and 200 µm-thick ZnTe (red line). The grey zone highlights the range of the spectrum where it is difficult to detect emission with EOS technique, and where THz QCLs typically operate.

Although a range of coherent detection methods exist and are being investigated [13], the key approaches have been electro-optic sampling (EOS) and photoconductive antennas (PCAs). EOS is based on inducing a birefringence by the Pockels effect on a second-order non-linear crystal [14]. Currently, it is the most commonly used technique, as it offers ease-of-use and does not require any device fabrication [15]. However, phase matching and phonon absorption in EO crystals (beyond 4 THz for typical non-linear optical crystals such as ZnTe [16]) drastically limits the useful bandwidth detected to a few THz [13] for a 800 nm, 100 fs detection beam. Although thinner nonlinear crystals can be chosen to extend the spectral bandwidth, this comes at the cost of a reduced THz signal due to a shorter interaction length. For instance, Figure 1 compares two ZnTe crystal thicknesses used for EOS in (a) the time domain and (b) the spectral domain. The grey area highlights the spectral range where emission becomes difficult – or even impossible – to detect with EOS, and where the best-performing THz QCLs typically operate.

**Figure 2:**
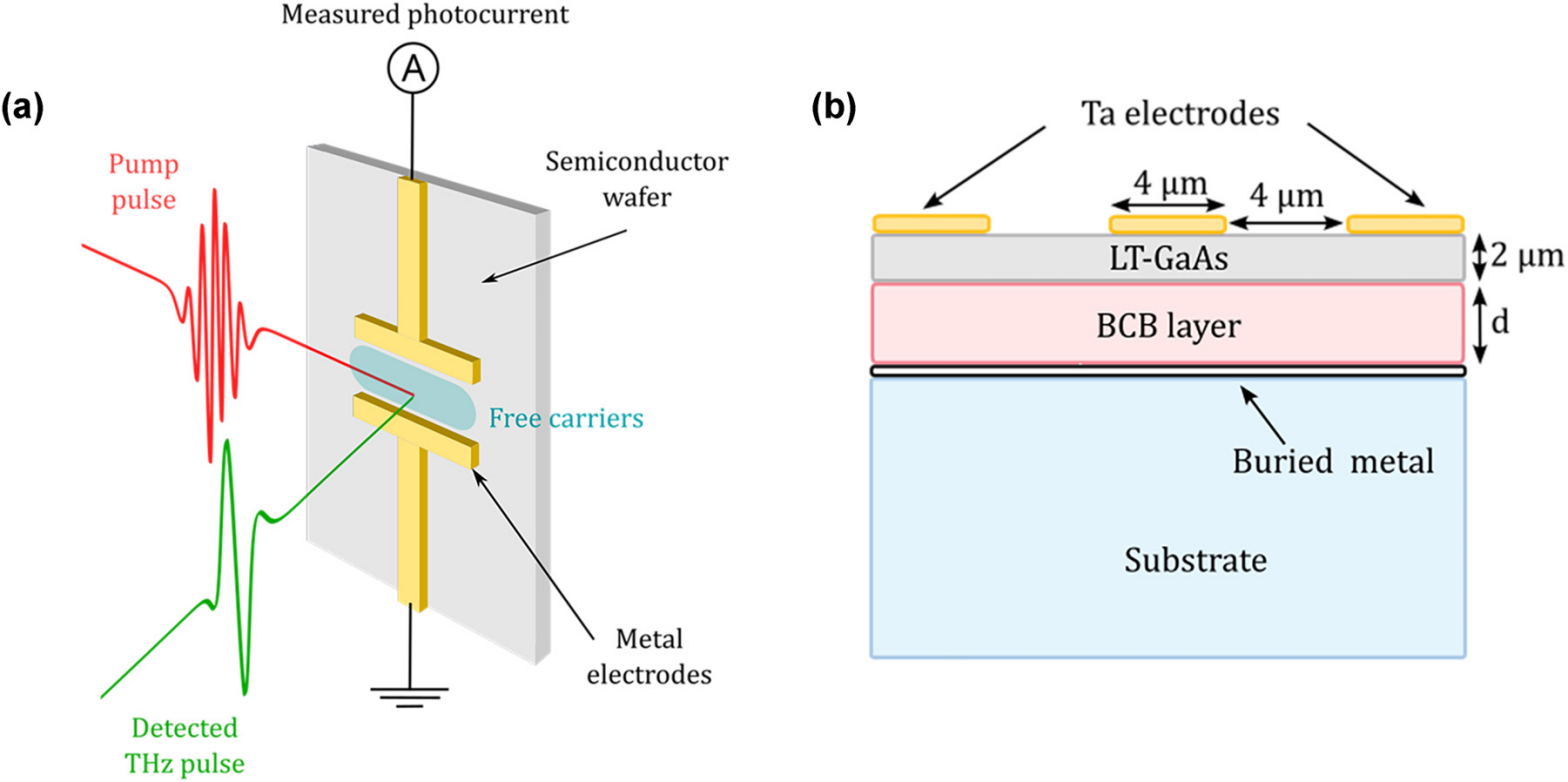
Schematic of PCA and cavity-based PCA detector. (a) Operational principle of a photoconductive antenna used to detect a THz pulse. Metal electrodes are deposited onto a semiconductor substrate. When the probe IR pulse (red pulse) arrives at a certain time on the substrate, it creates free carriers in the semiconductor, which are accelerated by the amplitude of incident THz field (green pulse) at the exact same time. This acceleration generates a photocurrent proportional to the THz field amplitude, which can be collected at the electrodes. By controlling the time of arrival of the IR pulse using a delay line, one can reconstruct the entire electric field temporal shape. (b) Structure of the THz-cavity antenna with a buried metal layer creating a cavity composed of a 2 µm LT-GaAs layer and a tunable polymer (BCB) layer of thickness *d*.

Regarding PCAs, these can also give a useful bandwidth from 0.1 to 4 THz, do not require a long interaction length [17] and have a similar operational principle for THz emission and detection. PCAs are based on ultrafast changes of surface photoconductivity of a semiconductor under femtosecond laser excitation. The devices are usually composed of two electrodes spaced by a certain gap and deposited on a semiconductor surface. When used as a detector, the optical excitation of the semiconductor gap, creates a sharp increase of free carriers that are accelerated by an incident THz electric field *E*_THz_, generating a time varying photocurrent *J*(*t*) (see Figure 2(a)) [18], [19] that is detected by the deposited electrodes. The PC antenna then works as an integrating detector:

(1) $$J\left(t\right)=e\mu \overset{\infty }{\underset{-\infty }{\int }}N\left(t^{\prime }-t\right)E_{\text{THz}}\left(t^{\prime }\right)\;dt^{\prime },$$

where *e* is the electron charge, *μ* denotes the electron mobility in the semiconductor, and *N*(*t*) is the total number of photo-generated carriers. With the acquisition of the photocurrent over time, one can access the time-resolved electric field profile [13]. In the case of an ‘ideal’ PCA with ultrashort carrier lifetime, the photocurrent measured is closely linked to *E*_THz_ as *J*(*ω*) ∝ *E*_THz_(*ω*), whereas with a PC antenna substrate with long carrier lifetime, such as GaAs, the induced photocurrent is *J*(*ω*) ∝ *E*_THz_(*ω*)/*ω*. One of the main limiting factors in ultra-broadband detection is thus the carrier lifetime in the semiconductor substrate and the sensitivity at higher frequencies is expected to decrease drastically with long carrier lifetimes such as in semi-insulating GaAs. However, when low temperature GaAs (LT-GaAs) is used, carrier lifetime is decreased drastically, allowing an improved spectral response. Note that carrier lifetime isn’t the only factor for broadband detection as the femtosecond pulse duration and the antenna geometry play also an important role [20], as well as the efficiency of *E*_THz_ collection. Nonetheless, in general and similar to nonlinear crystals, the spectral response drops rapidly beyond 1 THz limiting the bandwidth to a few THz. As QCL sources predominately operate at higher THz frequencies, the need for sensitive coherent detectors at frequencies higher than 3 THz has become an important technological goal.

Different methods have been used to extend the PCA detector bandwidth, such as shorter optical pump pulse duration (to 10 fs) [16], materials with shorter carrier lifetimes, such as low temperature grown GaAs (LT-GaAs), implementing antennas arrays [21] or designing nano-structured plasmonic electrodes [22], [23], [24]. These approaches extend the spectral bandwidth or increase the THz signal, keeping an extremely broad THz response. However, for THz QCL detection a larger bandwidth is not necessarily the required target but a larger response at a targeted frequency range around the QCL frequency.

As broadband detection is not targeted, here we build a THz cavity that permits to enhance the spectral response of a PCA over a desired spectral range at high THz frequencies, reducing the response at lower frequencies that are not relevant for QCL detection. In detail, we present a study aimed at enhancing THz detection with PCAs at higher frequencies ( > 3 THz), despite using a 100 fs laser for detection. The approach is based on realising a vertical THz quarter-wavelength cavity with a buried metal reflector [25] and a LT-GaAs active material [26], with the former permitting to enhance the spectral response at a targeted range and the latter allowing THz detection owing to its ultrafast carrier lifetime and high resistivity.

Although previous work has shown quarter-wavelength THz cavities for coherent THz pulse generation and detection [26], the spectral detection response was limited to low THz frequencies. This was a result of a 2 µm thick buried LT-GaAs layer on top of which was grown a normal GaAs spacer layer at a higher temperature, affecting the spectral performance of the LT-GaAs layer. Here, a new approach is based on transferring an as-grown LT-GaAs to a metal coated substrate with a low loss polymer spacer – benzocyclobutene (BCB) (see Figure 2(b)). The BCB layer presents the major advantage of being relatively tunable in its thickness via spin coating. It can therefore be used to change the total THz cavity size without requiring epitaxial growth. In this work, we detail the realisation of such a device, highlighting the fabrication process, its characterization as a THz detector and the comparison to EOS detection. The THz cavity also permits the planar electrode geometry to be engineered independently and we show that the cavity can be combined with large surface area single contact electrode to further enhance the spectral response. The electrode is based on an integrated resistance design allowing a better photocurrent distribution over the surface [24]. Despite the BCB layer not being fully optimised for the best response at 3 THz, the considerably increased THz spectral response can still be used to successfully demonstrate the coherent detection of a THz QCL operating at 3 THz, where a standard PCA does not show a response.

## 2 Design and fabrication

**Figure 3:**
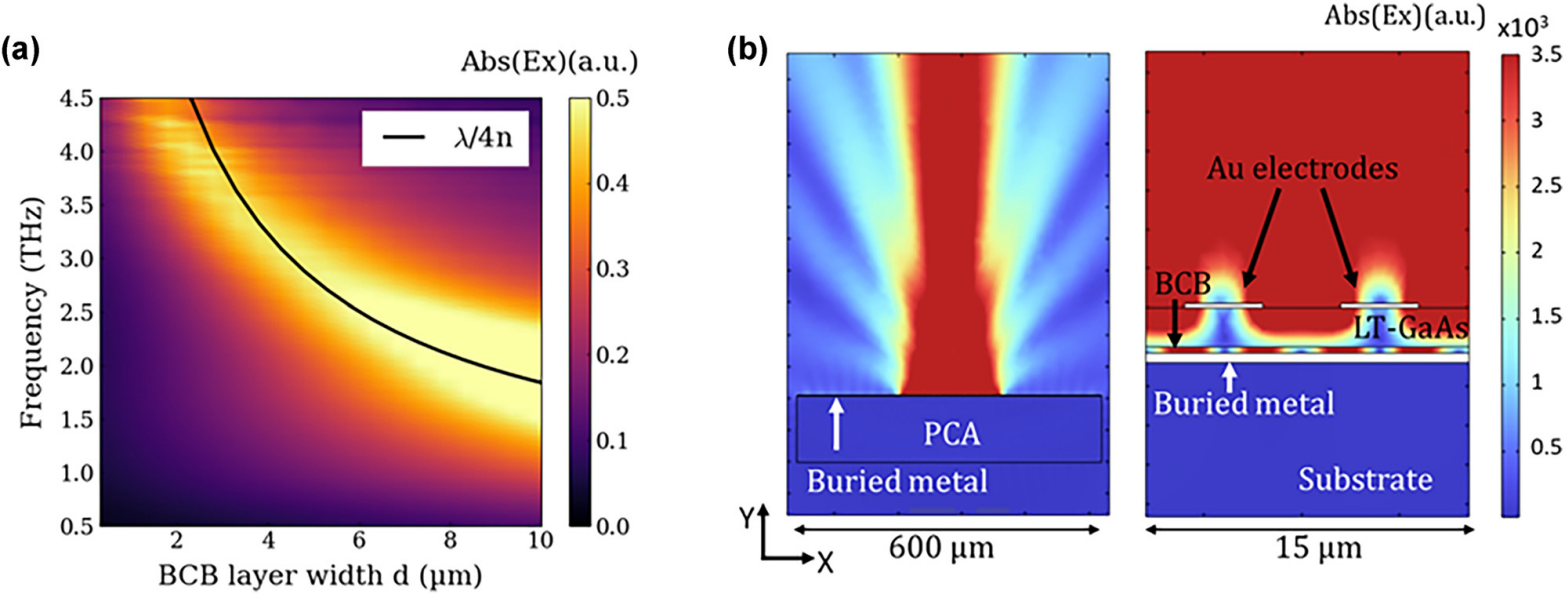
Electromagnetic simulations of THz cavity effect. (a) 2D plot of simulated electric field as a function frequency (*y*-axis) and BCB thickness (*x*-axis). The colour scale corresponds to the THz electric field amplitude (b) electromagnetic simulations of emitted field from cavity-based PCA with 0.3 µm-width at 3.5 THz. Right panel is the global electric field emitted by the entire structure, with the 200 µm long active region in the middle. Left panel is a zoom of the two electrodes and the mode confinement assured by the cavity created with buried metal.

To highlight the role of the cavity corresponding to a metal layer and polymer spacer below an LT-GaAs layer, the structure has been simulated in COMSOL Multiphysics by solving the propagation of the THz electric field generated by the cavity-based PCA [27]. A 2D model of our geometry coupled with the relevant material characteristics was build and solved using the finite difference time domain (FDTD) approach. An electromagnetic field probe is positioned above the structure to measure the relative electric field in reflection configuration, in the far field. The influence of the buried metal depth on the emitted electric field intensity at different frequencies is illustrated in Figure 3(a). This figure presents a colour map of the integrated field radiated by the device as a function of frequency (*y*-axis) and BCB thickness (*x*-axis) (measured from the bottom of the GaAs layer to the buried metal). The peak emission frequency shifts to higher values as the BCB thickness decreases, revealing a resonant behaviour. The black line added corresponds to the quarter-wavelength condition [28], [29], approximated as:

(2) $$\nu =\frac{c}{4\left(d+e_{\text{LT}-\text{GaAs}}\right)\left(n_{\text{LT}-\text{GaAs}}\cdot x_{\text{LT}-\text{GaAs}}+n_{\text{BCB}}\cdot x_{\text{BCB}}\right)}$$

with *x*_LT-GaAs_ and *x*_BCB_ being the fraction of the LT-GaAs and BCB layer thicknesses, respectively in regard to the total cavity thickness, which is *d* + *e*_LT-GaAs_, with *e*_LT-GaAs_ being the 2 µm thickness of LT-GaAs layer. *n*_LT-GaAs_ and *n*_BCB_ are the refractive indices of LT-GaAs and BCB, respectively. The frequency-dependent index of GaAs is used for *n*_LT-GaAs_ [30] and taken as constant for BCB (*n*_BCB_ = 1.8) [31].

The work here presents such a device with a 2 µm thick LT-GaAs and a 300 nm thick BCB spacer layer, shown schematically in Figure 2(b). This thickness of BCB was chosen as it can be realised in a single spin-coating step during processing: Although not of optimum thickness for detection around 3 THz, it demonstrates the first proof-of-concept as well as showing enhanced spectral response at high THz frequencies. Indeed, as seen in Figure 3(a), the optimised cavity size lies around 7 µm (5 µm BCB and 2 µm LT-GaAs). This will constitute the focus of further studies, realising thick BCB layers that will require optimisation of the BCB dilution, spin-coating characteristics and multiple layers to reach thicknesses of several microns, while also optimising the wafer transfer of LT-GaAs onto these thick BCB layers. Figure 3(b) shows the electric field generated by a 2.3 µm-cavity-size PCA and the mode confinement in the cavity.

**Figure 4:**
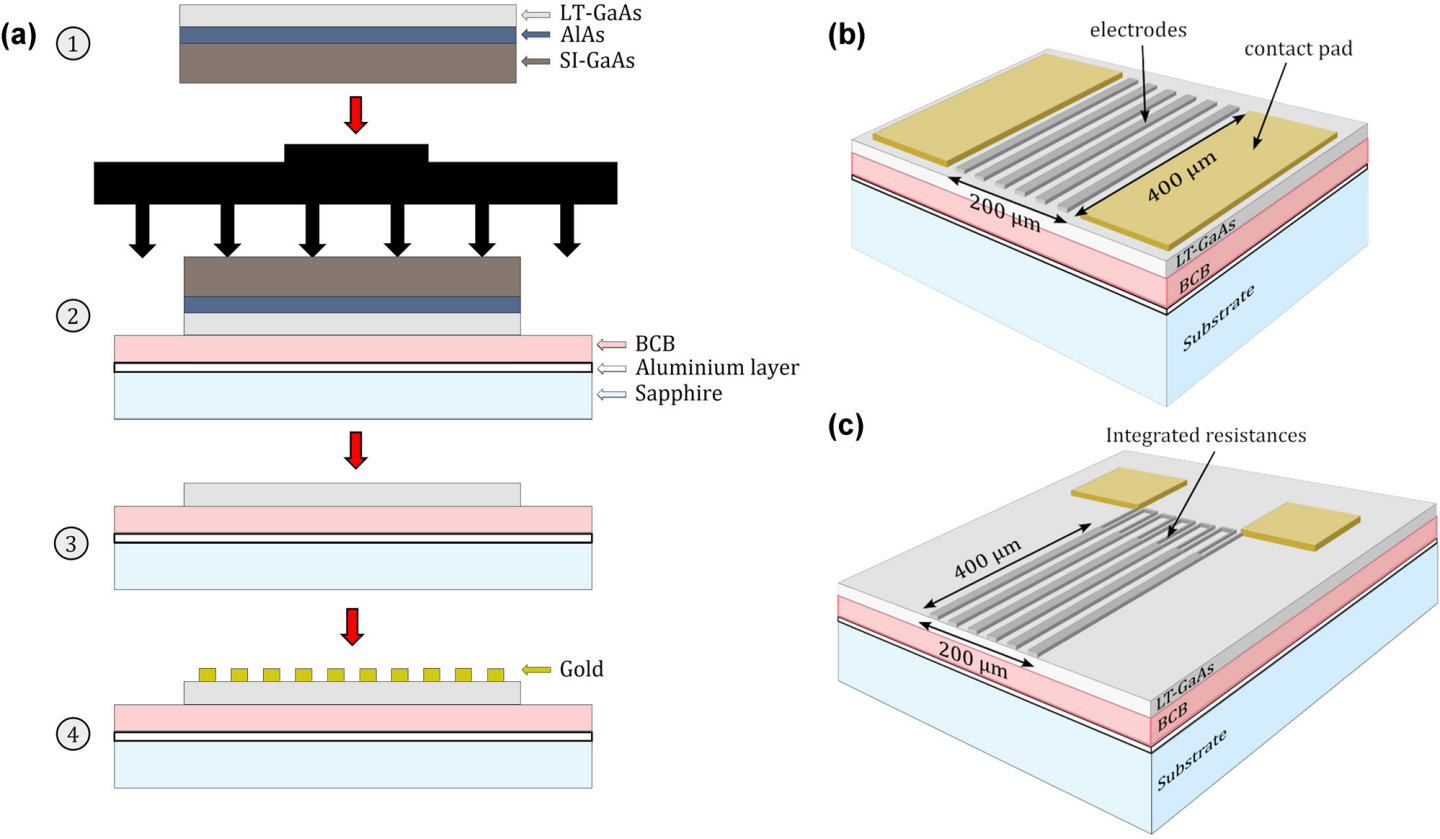
Fabrication process of cavity-based PCA. (a) Fabrication steps and layer bonding used for the realisation of the cavity based PCA detector: LT-GaAs is grown on an AlAs/SI-GaAs substrate and annealed at 510 °C for 15 min (step 1), then placed with tweezers on a sapphire substrate coated with an aluminium layer (metal mirror used for cavity) and a 300 nm-thick BCB polymer. The stack is compressed in a mechanical press and hard cured at 250 °C for 2 h (step 2). The AlAs layer and SI-GaAs substrate are removed by a combination of mechanical polishing and chemical etching (step 3), leaving the structure exposed for electrode deposition using photolithography (step 4). Two different large area electrode structures have been studied: (b) electrode structure with metal strips and (c) electrode structured with connected metal strips with loops for integrated resistances. The electrode area is approximately 200 μm m by 400 µm. Each electrode is of 4 µm width and separated by 4 µm gaps.

The sample was grown by molecular beam epitaxy and prepared using a wafer transfer technique [32]: A 2 µm thick LT-GaAs with an AlAs etch stop (100 nm-thick) was grown on a 500 µm-thick SI-GaAs wafer and then annealed at 510 °C for 15 min [33]. It was then mechanically transferred to a sapphire substrate coated with an aluminium layer (Al) (thickness 100 nm) and a 300 nm thick BCB layer (Figure 4(a)). The Al layer acts as the metal reflector. The host SI-GaAs substrate is then removed by mechanical polishing and two chemical etchings (citric acid followed by buffered HF), leaving the LT-GaAs exposed. The digitated metal electrodes are finally deposited by standard photolithography. The metal used is tantalum (Ta) which presents the advantage of having high resistivity [24], permitting the possibility of integrated resistances. This geometry was chosen to show that the planar electrode geometry can be combined independently with the vertical cavity. Large area structures of approximately 200 µm by 400 µm, with the digits being 4 µm wide with gaps of 4 µm, were realised for efficient detection of the THz pulses. Two electrode geometries were investigated with the first based on large gaps with unconnected high resistivity metal strips (see Figure 4(b)) and the second based on similar metal strips but connected in loops (Figure 4(c)) to realise integrated resistors [24]. This permits a more uniform distribution of the electric field across the entire structure, the different electrodes, as well as simpler processing compared to interdigitated antennas.

## 3 Characterisation

The samples were studied using a standard THz-TDS system based on a Ti:Sapphire 100 fs oscillator in reflection geometry. To characterize the detection process, a standard interdigitated PCA was used as an emitter with a quasi-static bias field (square wave, 2 V peak-to-peak at 17.7 kHz). The THz pulse generated by this PCA is collected by a series of parabolic mirrors and focused on the new cavity based PCA characterised with the optical femtosecond probe beam for THz detection. The THz-induced photocurrent is amplified by a transimpedance amplifier (gain of 10^8^) and measured using a lock-in amplifier with the reference frequency from the pulse generator of the generating PCA. To prevent water vapour absorption of the ambient atmosphere, the entire THz radiation path is purged with dry air. Once the time-resolved electric field is reconstructed with a mechanical delay line, a Fourier transform (FT) is applied to obtain the frequency spectrum.

A range of studies on the cavity-based PCA have been conducted. The first part shows the influence of the buried metal layer, emphasizing the effect of the cavity. The second part examines the impact of electrode structures by testing two different electrode designs (see Figure 4(b) and (c)). Finally, a preliminary comparison with the EOS method is presented in detail.

### 3.1 Influence of quarter-wavelength THz cavity

**Figure 5:**
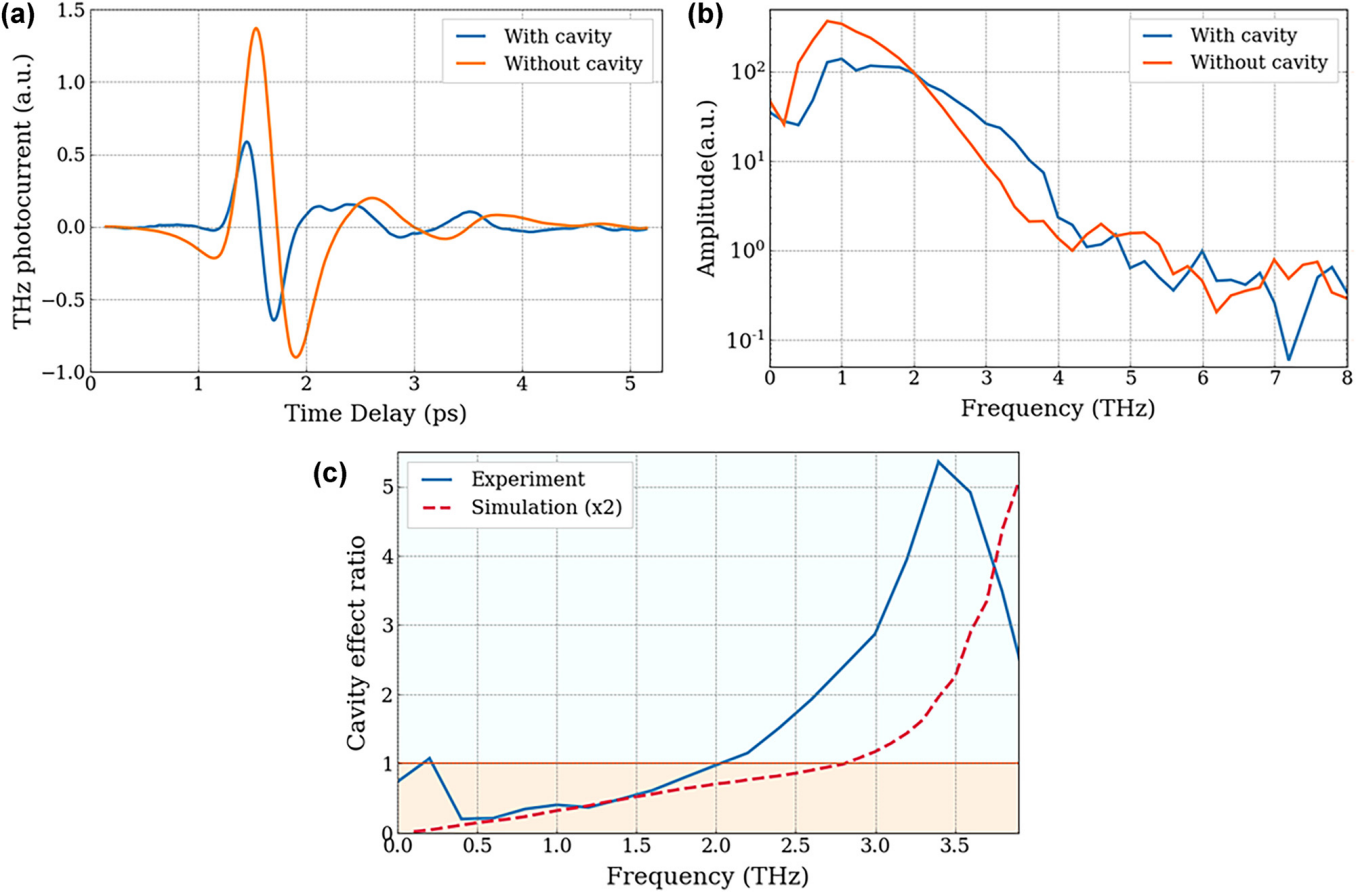
PCA based THz coherent detection with and without THz cavity. (a) Coherent THz pulse detected by the PCA structured without resistance loops with (blue line) and without (orange line) a THz cavity. (b) Spectrum corresponding to the FT of the time resolved electric field. (c) Effect of the THz cavity, characterised by the ratio of the spectrum with cavity to that without from experimental measurements (blue line) and COMSOL Multiphysics simulations (red dashed line) multiplied by a factor of 2 for comparison purposes. The orange area indicates the region where the ratio is less than one, i.e., the spectrum without the cavity shows a larger response. Conversely, the light blue area corresponds to the frequencies where the spectrum with the cavity is larger.

To study the influence of the buried-Al layer that permits the realisation of a THz cavity, the results are compared to those of a PCA with the same electrode deposition (without integrated resistances, see Figure 4(b)) and the same bulk structure but without the buried metal. Figure 5(a) and (b) show the detected THz pulses by the two PCAs, and their frequency spectra, obtained by Fourier transform of the temporal trace, respectively. Although the peak-to-peak electric field is smaller for the cavity based PCA, owing to its thin BCB layer that is not optimised for low THz frequencies, it clearly shows a much broader spectrum than the standard PCA. The detection becomes more efficient with the cavity-based PCA at frequencies greater than 2 THz and can be further observed in the cavity effect ratio (see Figure 5(c)) that represents the ratio between the spectrum of the cavity-based PCA (Figure 5(b) – blue line) to that of the PCA without cavity (Figure 5(b) – orange line). Below 1, the orange area represents the frequency domain where the detected signal from the PCA without cavity is higher. However, above 1, in the light blue area, the spectrum of the cavity-based PCA is stronger, highlighting the effect of the cavity as a function of frequency. This shows that at frequencies from 2 to 4 THz, the cavity has a positive impact on the detected spectrum with an enhancement of a factor of 5 around 3.5 THz. The red dashed line represents the simulated spectral ratio for an emitting THz PCA multiplied by a factor 2 for comparison purposes, assuming that the emission is a direct inverse of the detection process. The multiplication factor can come from the approximative arbitrary value used for surface current densities added in the simulation to artificially create the THz emission. The cavity effect is also seen but is shifted towards higher frequencies and with a smaller enhancement. Regarding the former, in the experimental data, this can be a result of the frequency limit of the antenna used as emitter and/or the limit of the spectral bandwidth of the 100 fs excitation laser. This limits a reliable cavity effect ratio to approximately 3.5 THz as the signal drops rapidly at higher frequencies. There could also be an influence of using the GaAs refractive index for LT-GaAs in the simulations and where previous studies in the optical range have shown that the refractive index can vary as much as 0.25 between the two materials [34]. The reason for smaller enhancement than observed experimentally is not clear but could be the result of electrode structure acting as an antenna array [35].

### 3.2 Impact of the electrode structure

**Figure 6:**
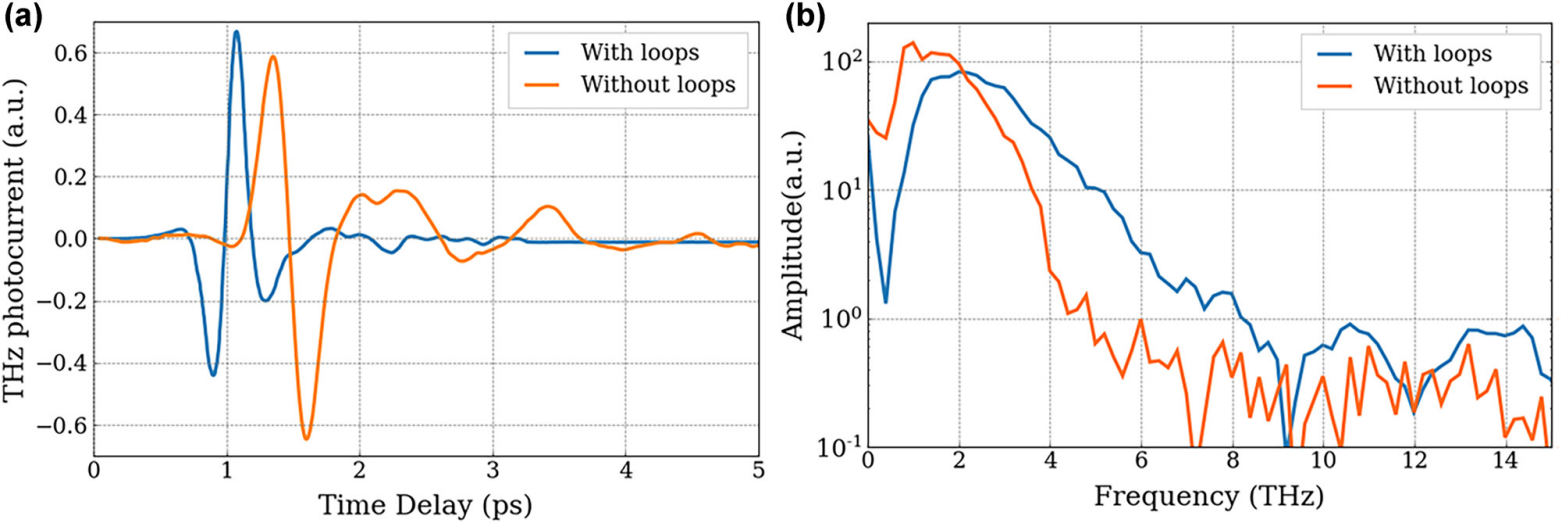
Influence of top electrode structure on THz detection. (a) Coherent THz pulse detected by cavity-based PCA structured with cavity with resistance loops (blue line) and without (orange line). (b) Spectrum corresponding to the FT of the time resolved electric field for a PCA with loops (blue line) and without (orange line).

To study the influence of the electrode structure with and without integrated resistance loops on cavity-based PCAs, the two schemes presented in Figure 4(b) and (c) were tested under the same conditions and the results are shown in Figure 6. A large increase is observed in the spectral response when using the sample with resistance loops. Indeed, as shown in Figure 6(a), whilst the time-resolved THz pulses are of similar amplitude, the spectrum has increased when integrated resistance loops were added, going up to 6 THz (see Figure 6(b)). This suggests an enhanced collection of the photocurrent over the entire surface of the PCA, compared to a standard large gap PCA where the generated current is too far from the anode to be efficiently collected before carrier recombination [22].

### 3.3 Comparison to EOS

**Figure 7:**
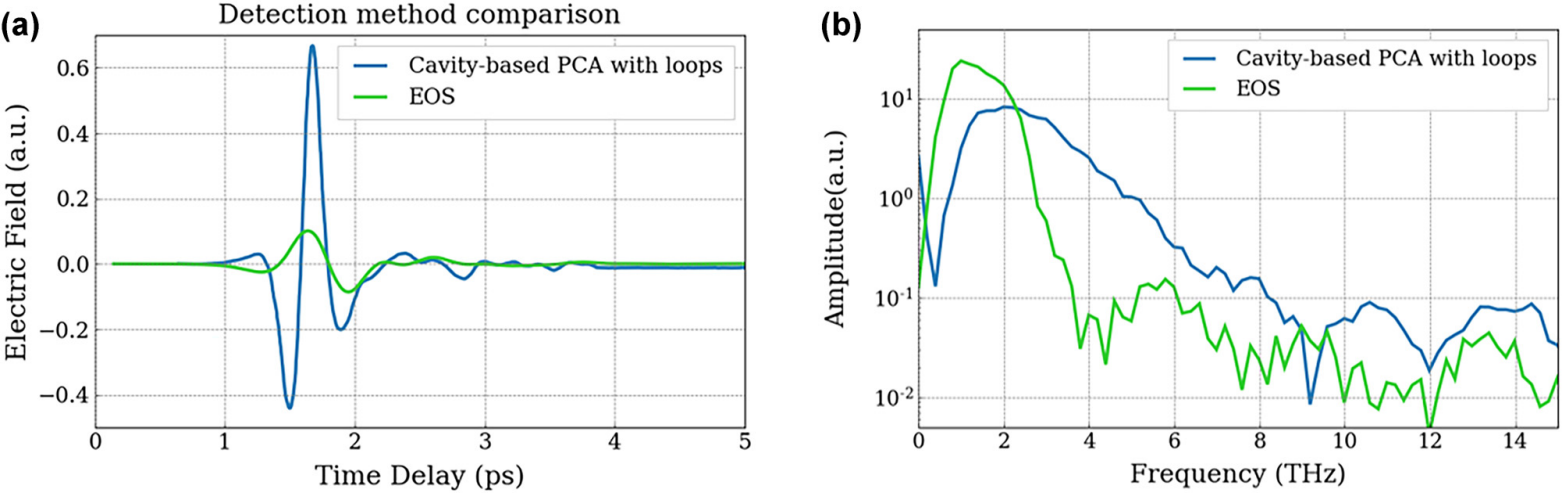
THz detection – comparison between cavity-based PCA and EO sampling. (a) Comparison between a coherent THz pulse detected with electro-optic sampling (green curve) and with a cavity-based PCA (blue curve). (b) Spectrum corresponding to the FT of the time-resolved electric field. The spectrum for the cavity-based PCA (blue curve) has been reduced by a factor of 10 in order to match the noise floor with that of EOS detection to compare the dynamic range.

The cavity-based PCA detector with integrated resistance loop electrodes (Figure 4(c)), which showed the most enhanced spectral bandwidth was compared to EOS using a thin 200 µm thick ZnTe crystal (Figure 7). Although the emitted electric field profile (Figure 7(a)) demonstrates significant improvement compared to that detected with EOS, a direct comparison between the two signals is not entirely possible owing to differing amplification factors. However, their dynamic range in the spectral domain can be meaningfully compared with a much broader spectral response for the PCA (see Figure 7(b)). Their spectra have been normalised to the noise floor to compare them properly where the cavity-based PCA spectrum is divided by a factor of 10. The signal-to-noise ratio of the PCA detector around 3 THz is approximately 10 times higher than that of EOS. (Note that no time echoes are observed for the PCA detector compared to EOS as seen in Figure 1).

## 4 Coherent detection of double metal QCL

**Figure 8:**
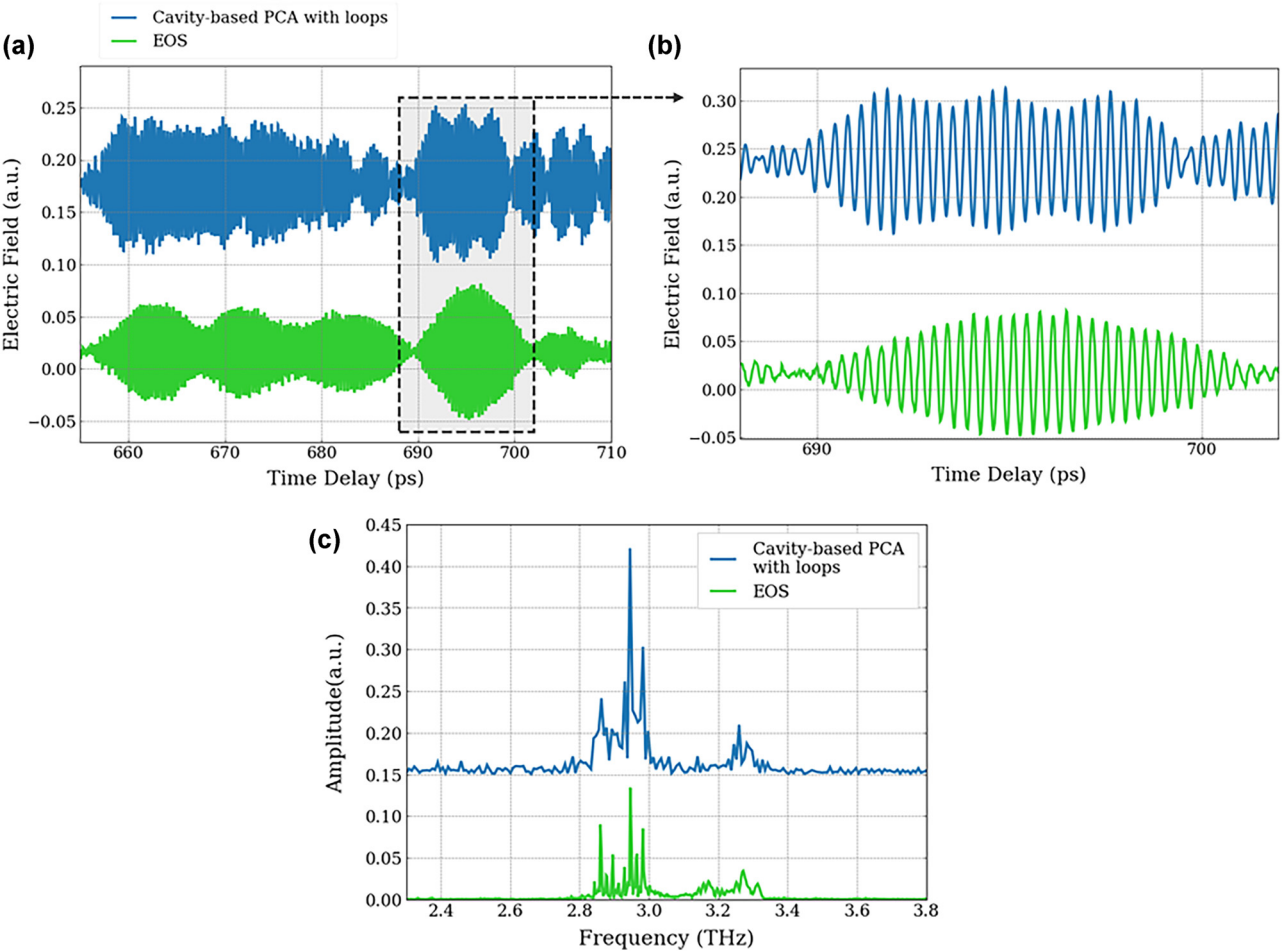
Coherent detection of THz QCL emission using PCA. (a) Comparison between the time-resolved THz electric field profile emitted by a double-metal QCL operating around 3 THz over a 55 ps time window. (b) Zoom of periodical oscillations of 320 fs emitted by the QCL. (c) Spectrum corresponding to the Fourier transform of the time resolved electric field. Green and blue curves correspond to detection with EOS and cavity-based PCA, respectively.

This enhanced detection at higher THz frequencies was then used to study coherent detection of a double-metal THz QCL with a hybrid design bandstructure. The QCL operates between 2.8 THz and 3.4 THz with the details of the growth given in Ref. [10]. An injection seeding approach is used, where the QCL demonstrates laser action on a coherent THz pulse that is coupled into the cavity of a gain-switched QCL, rather than on the QCL’s spontaneous emission. This then permits the coherent time-resolved detection of the QCL emission using coherent sampling techniques [4]. The THz radiation emitted by the QCL is collected by a set of parabolic mirrors and focused onto the coherent detection scheme with the femtosecond probe pulse. The two detection methods – cavity-based PCA and EOS – have been compared with the first measurements shown in Figure 8(a). This was taken at long times (∼630 ps) after the injection of the THz pulse (0 ps) where the QCL has been seeded by the injected pulse. The temporal scan, Figure 8(a), is a 55 ps scan showing a similar response between EOS and PCA detection and Figure 8(b) shows an enhanced view highlighting clearly time oscillations of a period of about 320 fs corresponding to the QCL emitting with a frequency distribution from 2.8 to 3.4 THz (see spectra in Figure 8(c)). Both detection methods show equivalent results. However, the signal-to-noise ratio of the PCA is less than that of EOS, which is possibly owing to electronic pick-up from the additional kHz and RF modulation for the QCL [4], which are used to coherently detect the QCL time-resolved emission. Although the overall response between EOS and PCA is similar, they are not exactly the same over the entire scan. It could be a result of the presence of higher noise measured for the PCA, the result of the large spectral response or the fact that in the case of the PCA there are no temporal echoes owing to no propagating pulses in the cavity [25]. Nevertheless, it should be noted that this is the first demonstration of coherent detection of double-metal QCL using a PCA, which further has not been optimised for detection at 3 THz and is expected to be enhanced by a factor 6 with the optimised cavity size.

## 5 Conclusions

By leveraging the flexibility of III–V material device fabrication, this work has demonstrated coherent THz detection using large-area, cavity-based PCAs, with a 2 µm thick LT-GaAs layer transferred onto metal-coated substrates via a polymer spacer. A first proof-of-concept device with a 2.3 µm cavity was studied, and its potential improvement towards resonance at 3 THz using a 7 µm cavity was discussed. Despite being a preliminary prototype, the cavity-based PCA detector showed a broad spectral response and an enhanced signal-to-noise ratio around 3 THz when compared to coherent detection using nonlinear crystals – specifically, a 200 µm thick ZnTe crystal, commonly used as a compromise between THz amplitude and bandwidth. After comparison between PCAs with and without a THz cavity, and the role of the large area surface electrode structure, these PCAs where used to coherently detect the temporal response of a double-metal THz QCL operating around 3 THz with an emission bandwidth of approximately 600 GHz. This is the first demonstration of using THz cavity-based PCAs to study the time domain response of double-metal THz QCLs, despite the fact that this first proof-of-principle demonstration has not been fully optimised. Indeed, by simply tuning the BCB layer thickness using spin coating, one can design and realise any arbitrary vertical cavity thickness, which can be exploited to tune the detector resonance to the one needed. For example, a BCB thickness of 5 µm would permit an enhancement of the THz spectral response by a factor of 6 for an electric field at 3 THz, ideal for the study of the coherent response of THz QCLs. It would also permit insights into mode-locking and frequency comb operation, harmonic generation [36], as well as potentially high quantum efficiency to investigate THz quantum correlations between QCL modes [37].
